# Fetoscopic Endoluminal Tracheal Occlusion-Synergic Therapies in the Prenatal Treatment of Congenital Diaphragmatic Hernia

**DOI:** 10.3390/ijms26041639

**Published:** 2025-02-14

**Authors:** Zsolt Bara, Horea Gozar, Nándor Nagy, Simona Gurzu, Zoltán Derzsi, Timea Forró, Evelyn Kovács, Ioan Jung

**Affiliations:** 1 Department of Pediatric Surgery and Orthopedics, George Emil Palade University of Medicine, Pharmacy, Science and Technology of Targu Mures, 540142 Targu Mures, Romania; zsolt.bara@umfst.ro (Z.B.); zoltan.derzsi@umfst.ro (Z.D.); 2 Clinic of Pediatric Surgery and Orthopedics, Targu Mures, County Emergency Clinical Hospital, 540136 Targu Mures, Romania; evelynkovacs@yahoo.com; 3 Doctoral School of Medicine and Pharmacy, George Emil Palade University of Medicine, Pharmacy, Science and Technology of Targu Mures, 540142 Targu Mures, Romania; forro.btimea@gmail.com; 4 Department of Anatomy, Histology and Embryology Semmelweis University, Tűzoltó Street 58, H-1094 Budapest, Hungary; nagy.nandor@semmelweis.hu; 5 Department of Pathology, George Emil Palade University of Medicine, Pharmacy, Science and Technology of Targu Mures, 540142 Targu Mures, Romania; simonagurzu@yahoo.com (S.G.); janos.jung@umfst.ro (I.J.); 6 Romanian Academy of Medical Sciences, 030173 Bucharest, Romania

**Keywords:** congenital diaphragmatic hernia, fetoscopic endoluminal tracheal occlusion, materno-fetal, preterm birth, lung development, regenerative medicine

## Abstract

Congenital diaphragmatic hernia (CDH) is a relatively rare and severe developmental disease. Even with the most recent multidisciplinary therapies, the risk for neonatal mortality and morbidity remains high. Recent advancements in prenatal treatments, alongside experimental and clinical data, suggest that fetoscopic endoluminal tracheal occlusion (FETO) promotes lung development and offers a promising strategy against lung hypoplasia and pulmonary hypertension. It is the only existing direct mechanical therapy that intervenes in the regulation of pulmonary pressure. Its influence on lung development also interferes with tissue homeostasis and cell differentiation; it also enhances inflammation and apoptosis. Its physiopathology on cellular and molecular levels is still poorly understood. Unfortunately, the procedure also carries significant pregnancy-, maternal-, and fetus-related risks. Assessing a multifaceted intervention requires a collective view of all aspects. This scoping review uncovers potential materno-fetal procedure-related risks and highlights innovative solutions. Future research on lung development therapies in CDH may focus on the “dual hit” mechanism, combining molecular-targeting drugs and regenerative medicine with the mechanical nature of FETO for synergistic effects.

## 1. Introduction

Congenital diaphragmatic hernia (CDH) is a relatively rare developmental condition that occurs in about 1 out of every 2500 live births [1]. Approximately 85% of the defects are left-sided [2]. The herniation of abdominal organs through the diaphragmatic defect into the thorax occupies space and hinders prenatal lung development [3]. The impaired growth leads to pulmonary hypoplasia, characterized by fewer distal airways, reduced size, thickened alveolar walls, and increased volume of the interstitial tissue [1]. Additionally, external compression inhibits pulmonary blood vessel maturation, resulting in a reduced density of the lungs, altered responsiveness and increased muscularization of the pulmonary vessels, and modified molecular signaling; all these factors contribute to the development of pulmonary arterial hypertension [4]. These significant morbidities are linked with high neonatal mortality.

Prenatal diagnosis is achieved in two-thirds of cases [5,6], allowing for the severity assessment of the condition before birth [7]. The survival rates in severe cases of left-sided CDH are less than 25% after birth [8,9].

Although diaphragmatic herniation can be surgically corrected, herniation of the viscera in utero may lead to irremediable consequences. Accordingly, a great interest has arisen regarding the in utero treatment for CDH. A minimally invasive technique was developed after the realization that numerous invasive and risky prenatal interventions had not yielded clinically promising results [10]. Fetoscopic endoluminal tracheal occlusion (FETO) is a procedure that involves placing an inflatable balloon into the fetal trachea using a one-port fetoscope. The occlusion causes the accumulation of fluids, higher airway pressure, and cellular proliferation in the fetal lungs, improving the alveolar airspace and maturation of the pulmonary vasculature [11,12]. Experimental and clinical evidence indicates that FETO improves the lung size, and thus, neonatal outcomes [13]. Since FETO has become accessible, adequate patient eligibility assessment for the procedure has become vital [14]. The TOTAL trials demonstrated an increase in the survival rates by 25% in fetuses with severe CDH and by 13% in those with moderate CDH at discharge from the neonatal intensive care unit [15,16]. Furthermore, results from several centers worldwide sustain the procedure’s positive impact on the outcome [13,17,18,19].

The “dual-hit” hypothesis of CDH defines two types of insults to pulmonary development. Initially, before diaphragm formation, both genetic and environmental factors can alter both lungs. Later, the herniating abdominal organs and their development in the thoracic cavity mechanically affect the ipsilateral lung. This physical push also interferes with fetal breathing movements [20,21], which usually causes the stretching of developing airspaces and promotes growth. Lung development combines mechanosensitivity, fine mechanotransduction, and several molecular processes. FETO is currently the only existing and effective mechanical therapy; it directly intervenes in regulating pulmonary pressure and the transpulmonary pressure gradient [22].

FETO poses significant risks to pregnancy, mothers, and fetuses, yet its underlying mechanisms on lung development are still mostly unknown. When assessing the effects of such a multifaceted intervention, it is essential to view all aspects collectively. This scoping review aimed to uncover all potential materno-fetal procedure-related risks, challenges, and pitfalls while highlighting the innovative solutions available for these concerns. We utilized the combination of specific search terms, such as “congenital diaphragmatic hernia”, “fetoscopic endoluminal tracheal occlusion”, “tracheal occlusion”, “prenatal treatment”, “fetal surgery”, “lung development”, “iatrogenic preterm prelabor rupture of membranes”, “ex-utero intrapartum therapy”, “extracellular vesicles”, “regenerative medicine”, and “molecular”. We included all studies regarding the impact of FETO on all aspects while excluding those related to CDH that did not pertain to the procedure.

**Figure 1:**
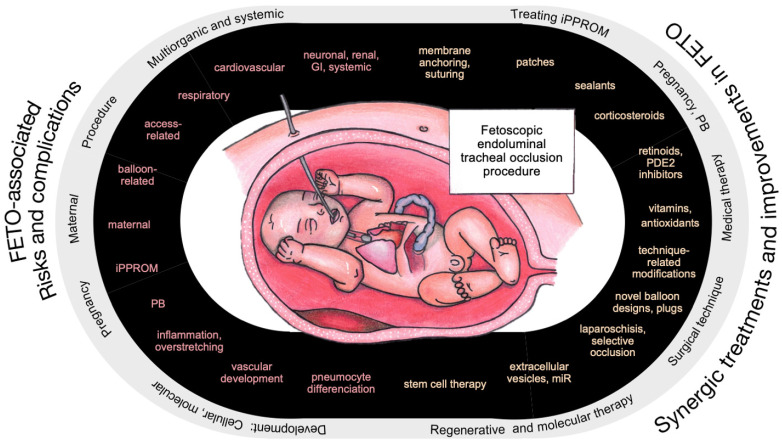
Risks and complications associated with fetoscopic endoluminal tracheal occlusion (FETO), and potential synergistic treatments and improvements. Abbreviations: iPPROM—iatrogenic preterm prelabor rupture of membranes, PB—preterm birth, GI—gastrointestinal, PDE—phosphodiesterase.

Future research on lung-development-improving therapies in CDH might rely on and start from the concept of the “dual hit” mechanism. Combining molecular-targeting drugs and regenerative medicine with the mechanical nature of FETO might result in synergy. Figure 1 represents the core of our paper, highlighting the risks and complications associated with FETO, as well as potential synergistic treatment options and ongoing advancements.

## 2. FETO-Associated Risks, Challenges, and Pitfalls

Fetoscopy is a microlaparoscopic technology used for fetal diagnosis and therapeutic interventions. It offers several advantages over traditional open fetal surgery, eliminating the morbidity associated with a large hysterotomy. Nowadays, its use is growing, becoming the most frequently applied technique for twin–twin transfusion syndrome; conditions such as urinary tract obstructions, cardiovascular anomalies, fetal tumors, and myelomeningocele; and for the prenatal treatment of CDH [19]. Although FETO is minimally invasive, it is still considered a risky procedure. It poses potential complications that extend beyond the general fetal-surgery-related ones, encompassing specific risks unique to FETO.

### 2.1. Procedure-Related/Technical Complications

Technically, the current FETO procedure uses a 3.3 mm cannula, which is introduced percutaneously under the guidance of ultrasound. An endoscope is then used to place an off-label applied endovascular balloon into the trachea, where it is inflated just below the vocal cords under direct visual control. The balloon has a one-way valve at its proximal edge to prevent deflation, and the internal pressure helps keep it securely in place during development. The produced lung fluids are trapped, leading to gradual lung expansion over time [23]. Balloon placement is usually performed between 27 and 29 weeks of gestation in severe cases and, later, at 30–32 weeks in moderate cases [24]. The benefits of using the plug–unplug sequence on cellular differentiation and maturation were proven. Then, a secondary step involving balloon removal is necessary around 33-34 weeks [25,26]. This can be performed in utero using either a fetoscope or ultrasound-guided puncture [27]. If these methods are not possible, ex utero intrapartum therapy (EXIT) must be applied. Usually, EXIT is applied for cases of congenital airway obstruction that pose a risk of asphyxia or neonatal death. This high-risk intervention involves delivering the fetus via C-section, while the placenta provides the respiratory support until the umbilical cord is clamped [28]. Regarding FETO, the procedure allows for the elective or emergency removal of the tracheal balloon via bronchoscopy.

Theoretically, maintaining the occlusion until delivery might result in additional lung growth and prevent the need for prenatal balloon removal. However, it could also lead to a higher number of emergency removals while still in placental circulation or even postnatally, which could be more challenging and potentially unsuccessful [27,29]. If the occlusion is reversed less than 24 h before delivery, the metallic balloon components are more likely to be retained [30]. Spontaneous deflation of the balloon is rare [31].

### 2.2. Pregnancy and Maternal Risks

Even though iatrogenic preterm prelabor rupture of membranes (PPROM) is less common in minimally invasive fetal procedures compared with open fetal surgery, it is still the most significant complication [32]. It occurs in approximately 30% of cases treated with minimally invasive fetal surgery. PPROM is defined as the rupture of membranes before the 37th week of gestation. Prematurity can be categorized into three groups: extreme (less than 28 weeks), moderate (28 to 34 weeks), and late (34 to 37 weeks) [33]. The literature has limited information regarding the risk factors and incidence of iatrogenic PPROM. When discussing PPROM, it is important to consider the abnormal nature of these pregnancies [34].

The instrument size is linked to the rate of iatrogenic PPROM, fetal survival, and gestational age at birth [32]. The cannula insertion site is also crucial. Introducing the cannula at the upper uterine position is more advantageous than the lateral lower sites [18,35]. Additionally, twin pregnancies are associated with at least twice the risk of PPROM compared with singletons [36].

Potential mechanisms associated with iatrogenic PPROM include postoperative chorioamnionitic separation, damage to port sites causing membrane apoptosis, membrane overdistension, dehydration, and amniotic fluid dilution [37]. Oligohydramnios or anhydramnios may be observed after PPROM. When vaginal fluid leaking is experienced, a “fern test” can be used to confirm its origin. If the leakage is intraperitoneal, the test result will be negative [38].

CDH pregnancies have been associated with up to 29% of cases of PPROM and are typically managed with expectant care [39]. Considering these statistics, the fact that 40–47% [40] of reported cases were managed with FETO suggests a clear impact of the procedure. However, this impact may not be as pronounced as it appears without a baseline comparison [34].

Related or not to PPROM, preterm birth (PB) is another associated issue defined as birth before the 37th week of gestation. Studies have reported that 70% of PBs occur before the 37th week, with 18% happening before the 32nd week [41]. PB is linked to poor pregnancy outcomes. Factors such as maternal age, ethnicity, socioeconomic status, and BMI are linked to PB; therefore, it is crucial to assess the risk of PB before starting any fetal therapy [42,43].

Lastly, fetal interventions do not exert any medical benefits to the mother. Ethically, maternal risks should be reduced to a minimum and be acceptable to both herself and her family. When considering the potential maternal complications that may arise from fetal surgery, several issues can occur, including hemorrhage and bleeding (e.g., from placental trocar placement, peri-incision, or hemoperitoneum), placental abruption, chorioamnionitis, endometritis, uterine rupture, pneumonia, pulmonary edema, sepsis, venous or amniotic fluid embolism, gastrointestinal bleeding, and wound-related complications. Some of these complications could be severe enough to require early delivery or even the termination of pregnancy at the time of the intervention [44].

To the best of our knowledge, there have been no maternal deaths associated with FETO [44]. Compared with the expectant management of CDH, fetoscopic procedures may not affect obstetrical outcomes or future reproductive potential. However, pregnancies complicated by serious congenital birth disorders, like CDH, can have significant psychological impacts [45].

### 2.3. Fetal FETO-Associated Lesions

Very few studies investigated the potential postnatal side effects of FETO, and even fewer included long-term follow-up cohorts.

Respiratory lesions are the most observed complication, with pulmonary hypoplasia being one of the main associated effects of CDH, as previously described. It is related to postnatal ventilation-induced lung injury, which further impairs respiratory function. Tracheomegaly and tracheobronchomegaly, which refer to an increased diameter of the trachea and bronchi, are common. This condition is often associated with a higher likelihood of tracheomalacia [30,46]. However, the size of the trachea does not seem to influence the survival rates or the necessity of early respiratory support [47]. Stridor, effort-induced barking cough, and recurrent chest infections may develop later. Among CDH survivors, the prevalence of chronic lung disease has been reported to be up to 50%, predominantly affecting intensive resuscitation and extracorporeal membrane oxygenation (ECMO)-requiring severe cases [48]. Stenosis or laceration of the trachea can occur, which may be severe and require tracheostomy, tracheal suturing, or stenting [49].

FETO may have a secondary effect on the growth of the left heart. Although fetal left heart hypoplasia may worsen during intervals, it tends to normalize after surgical repair. This could be due to the pulmonary venous return to the left heart and higher intrathoracic pressures following the procedure [50]. Postnatal assessments revealed enlargement of the left heart and the left pulmonary artery. These hemodynamic alterations might be related to changes in ventricular loading influencing heart growth [51]. However, another study indicated that there was no significant effect on the left ventricle size and function after the procedure.

Infants with CDH often require significant medical support, including ventilatory assistance, administration of inotropes, and potentially nephrotoxic drugs. It was suggested that infants treated with FETO present a higher risk of developing an acute kidney injury (AKI). AKI might be associated with increased mortality, lengthened mechanical ventilation, and longer hospital stays in these infants. In addition, AKI might be related to the severity of hypoxia and hypoxic respiratory failure. FETO-treated infants often present a moderate-to-severe spectrum of CDH. A higher oxygenation index has also been associated with an increased risk of AKI [52].

Gastroesophageal reflux disease (GERD) is one of the most common long-term comorbidities associated with CDH, observed in up to 81% of patients [53]. Due to the anatomical and functional respiratory changes that occur during and after FETO, GERD may be more severe in these patients. While further evidence is needed, FETO appears to reduce the herniation of abdominal organs and also gastric torsion, which may help limit the severity of GERD [54]. Additionally, FETO does not seem to affect the risk of gastrointestinal morbidity [55].

CDH survivors are also at risk for neurodevelopmental dysfunction, which is recognized as the most disabling outcome associated with CDH [56]. In comparison with severe CDH managed with expectant care, FETO appears to be linked to more favorable neurodevelopmental outcomes at 24 months [57].

A multidisciplinary prospective study showed improvements in pulmonary hypertension, morbidity, and GERD metrics. These results revealed favorable survival rates and morbidity with a median follow-up of 5.8 years [58].

### 2.4. Effect of FETO on Lung Development

Recent knowledge on the cellular and morphological mechanisms of FETO is partial and based mostly on experimental findings. Tracheal occlusion (TO) promotes lung growth; however, the developing lung also suffers excessive mechanical stretching, resulting in abnormal alveolar development and function [59,60]. The effects of TO are not homogenous, leading to different metabolic and morphologic responses within and between the two lungs, as demonstrated in a rabbit model of left CDH [61].

Low lung compliance in CDH can arise from several factors, including abnormal remodeling of the lung structure or primary surfactant deficiency. However, the latter is a controversial topic in CDH research [62]. The surfactant is composed of proteins and phospholipids secreted by alveolar epithelial type II (AE2) cells, which are the precursors of the elongated type I cells responsible for gas exchange. The surfactant is indispensable in reducing surface tension at the air–liquid interface of the alveoli [63]. Mechanical stretching promotes the growth and development of lung cells by influencing the expression of certain genes and the synthesis of extracellular matrix (ECM) components [64]. Inadequate lung expansion in CDH also impairs the AE cells’ differentiation. Experimental data indicate that the resultant lung hypoplasia leads to an increased AE2 cell density and overexpression of surfactant proteins [65]. However, it was observed that the levels of phospholipids in the bronchoalveolar lavage and surfactant proteins in lung tissue are reduced at birth [66]. Other studies challenged the notion of primary surfactant deficiency in CDH. They considered that the regulation of AE2 cell maturation and surfactant content were normal. Additionally, external factors, such as high-level inspired oxygen ventilation can also negatively impact postnatal surfactant synthesis. It is also noted that the degree of surfactant deficiency may correlate with the severity of pulmonary hypoplasia [62]. A current cohort indicates that the patients not treated with FETO but received surfactant therapy had worse outcomes. In contrast, the FETO population treated with a surfactant showed better survival rates and less ECMO use [67].

TO causes lung expansion, accelerates AE2 cell differentiation, and increases apoptotic activity [68]. Reversible or temporary TO reduces these adverse effects. It might positively affect the expression of various factors involved in pulmonary development, recovery of AE2 cells, and surfactant production [22]. However, the increase in lung compliance that is not related to surfactant mechanisms is more significant [68]. Compared with the non-reversed TO, the increase in alveolar wall thickness remains persistent [69].

CDH is linked to a reduced number of pulmonary arterial branches, exhibiting increased muscularization extension, along with greater media and adventitia thicknesses and an increased external diameter [70]. Clinically, these changes lead to pulmonary hypertension: the persistence of fetal circulation and the significant right-to-left shunt result in hypoxia, hypercapnia, and acidosis. In CDH, factors contributing to pulmonary hypertension may include the excessive development of pulmonary artery muscles, an underdeveloped pulmonary vascular bed, irregular expression of vasoconstrictors or relaxants, altered responses to vasoactive agents, and surfactant deficiency [25].

Experimental data show that short-term TO improves pulmonary hemodynamics by profound changes in the vascular bed structure. The muscularization of vessels does not pass the preacinar level: the increased medial wall thickness (MWT) is corrected at both the preacinar and alveolar levels. The mechanisms of pulmonary vascular maturation include endothelial activation of nitric oxide synthase and the expression of endothelins and their receptors [71].

In FETO-treated CDH lambs, the pulmonary growth correlated with the increased total pulmonary blood flow, suggesting the proportional development of the vascular bed with the lung. Despite the reduced pulmonary vascular resistance and improvements in respiratory mechanics, FETO did not enhance the gas exchange during the cardiopulmonary transition at birth. In this context, the lung-weight-adjusted pulmonary blood flow was not significantly different from that of the CDH lungs not treated with FETO [72].

### 2.5. Effects of FETO at Molecular Level

While FETO as a prenatal CDH therapy may gain acceptance, understanding the underlying cellular and molecular responses is crucial for developing novel adjuvant therapies that enhance fetal lung development and minimize adverse effects. It is worth mentioning that current evidence regarding the underlying molecular aspects of FETO mostly derives from animal studies that investigated the effects of non-reversed TO [73].

The mechanisms behind expansion-induced lung growth, cell differentiation, and tissue remodeling are still under investigation. However, FETO cannot be viewed as merely reversing the changes induced by CDH. It induces alterations in cellular function, which may have lasting consequences. A proteomic analysis of tracheal fluid revealed that TO-treated samples are far different from non-treated ones, with the latter being more similar to healthy controls. Important signaling pathways, such as transforming growth factor β (TGF-β) and mammalian target of rapamycin (mTOR), that were downregulated in CDH samples were upregulated in the TO-treated ones. TO activated the AKT-related signaling cascades: increased epithelial proliferating cell nuclear antigen (PCNA) and phosphorylated AKT. It also enhanced cilia-related pathways, like acetylated α-tubulin, and relatively increased the number of ciliated cells. Wnt-Axin2 signaling was not substantially reversed in these cases [74].

The expression of various growth factors is also believed to be crucial for lung growth and development. These include platelet-derived growth factor (PDGF), vascular endothelial growth factor (VEGF), insulin-like growth factor (IGF)-II, TGF-β [75], keratinocyte growth factor (KGF), fibroblast growth factor-10 (Fgf10), and epidermal growth factor receptor (EGFR).

Fgf10 is recognized as a key regulator of airway-branching morphogenesis [76]. In the pseudoglandular stage of murine lung development, TO leads to increased Fgf10 and phosphorylated extracellular signal-regulated kinase (ERK) levels and a decreased expression of Sprouty homolog 2 (Spry2). Notably, TO in FgfR2B-deficient lungs does not result in enhanced branching, confirming the pathway’s importance in this process. The Fgf10-FgfR2-Spry2 pathway is also activated in response to positive transpulmonary pressure [77]. Negative transpulmonary pressure decreases Fgf10 and Spry2 expressions, indicating that lung tissues can detect the pressure direction. Adding the Fgf10 to these tissues corrected branching defects, showing this growth factor’s crucial role in transmitting mechanical signals during early lung development [78].

KGF, a strong stimulant for AE2 proliferation and maturation, is an epithelial-specific growth factor primarily produced by mesenchymal cells, like vascular smooth muscle cells and fibroblasts [79]. KGF exerts a paracrine effect and, along with other growth factors, such as Fgf10 and hepatocyte growth factor, is crucial for lung development [80]. It significantly facilitates AE2 cells maturation and boosts the expression of phosphatidylcholine and surfactant proteins A, B, and C. Research indicated that KGF levels are reduced in both nitrofen-induced and surgical CDH models. It seems that TO restores normal levels of KGF expression [81,82].

EGFR is essential for the self-renewal of AE2 cells and plays a significant role in myofibroblast migration during alveolar septation [83,84]. In CDH, the EGFR and ephrin signaling pathways are downregulated. However, these pathways were found to be markedly elevated in TO-treated CDH rabbit pups. Activated by the mechanical stimulus of TO, small GTPases, like Csk/Src and Ras homolog family member A (RhoA), act through CDC42 and Rac1. EGFR may also be connected to this dysregulated signaling [73,84]. By identifying and blocking these ligands, we may enhance lung development by preventing epithelial hyperplasia, promoting epithelial maturity, and improving lung function [73]. This might be achieved by inhibiting tyrosine kinase activity or using a monoclonal antibody to deactivate the ligand or receptor [85,86].

TGF-β is another key factor in alveolarization and lung-branching morphogenesis [87]. Inferior lung tissue mechanics caused by TO appear to be linked to the increased transcription of ECM components. This rise in myofibroblast differentiation and matrix deposition may be associated with TO-enhanced TGF-β/Rho kinase pathway activation and elevated TGF-β transcript levels. Further pathway analyses indicated an increase in Rho-associated kinases, which negatively impacted Smad2/3 activation. After TO, the accumulation of α-smooth muscle actin and collagen was observed in the alveolar walls of rabbit and human CDH lungs that experienced short-term mechanical ventilation [88].

Yes-associated protein (YAP), a central mechanotransducer, seems to play essential roles in branching morphogenesis and alveolar epithelium differentiation. YAP interacts with TGF-β and Sox2, acting as a regulator in the formation of the pulmonary structure [89]. Impaired nuclear YAP activity is related to alveolar differentiation defects and represents a potential therapeutic target. Research showed that TO also normalizes the level and nuclear localization of YAP, and thus, epithelial differentiation [90].

VEGF, endothelins, neuregulin, and KGF seem to influence the pulmonary cellular and vascular development during TO [22]. VEGF is an angiogenic factor produced by AE2 cells that promotes the growth, proliferation, and angiogenesis of endothelial cells. In nitrofen rat models, VEGF levels and signaling are considerably lower [91]. This effect is rescued by TO, as marked by an increase in VEGF-A expression [92,93,94]. VEGF may contribute to the proliferation of endothelial cells triggered by TO. However, the underlying mechanisms that lead to the expansion-induced proliferation of AE cells and fibroblasts are probably not related to the upsurge in IGF-II or PDGF-B expression, nor to the activation of the MAPK pathway [75].

The role of nitrogen oxide (NO) synthase in pulmonary hypertension associated with CDH is still debated, and its effects are not fully understood. TO and pulmonary ventilation lead to changes in the NO pathway, resulting in decreased NO synthase expression in the pulmonary vessels of fetuses affected by CDH [95].

Inflammation can affect the expression of various growth factors that promote lung growth and inhibit certain genes involved in vascular proliferation, as well as structural and plasticity changes. Following TO, damage associated molecular patterns (DAMPs) release might occur due to several mechanisms, including cellular death, passive release into the extracellular space, active secretion triggered by cellular stress, or formation/activation of damaged ECM fragments. Various molecules, such as mitochondrial transcription factor A, high-mobility group box 1, heat shock proteins (HSP60, 70, 90), extracellular tumor necrosis factor (TNF)-α, interleukin (IL)-1β, adenosine triphosphate (ATP), reactive oxygen species (ROS), ROS-modified molecules, angiotensin II, and ECM degradation products can act as DAMPs. Some of them may interact with toll-like receptors in the fetal lung after TO [96].

Inflammatory pathways in CDH are also activated via signal transducer and activator of transcription 3 (STAT3) signaling and the upregulation of diverse circulating microRNAs (miRs) contributing to abnormal lung development. Several STAT3-related cytokines, such as IL-2, IL-9, and IL-15, were higher in tracheal aspirates of FETO-treated CDH survivor fetuses than in non-survivors [97].

**Figure 2:**
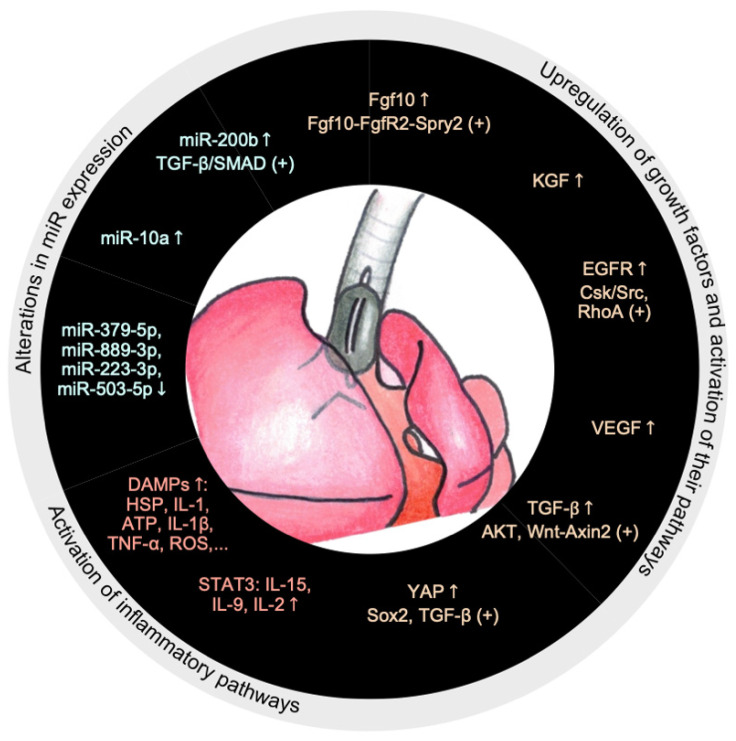
Main FETO-associated molecular alterations. Abbreviations: Fgf10—fibroblast growth factor 10, FgfR2—fibroblast growth factor receptor 2, Spry2—Sprouty homolog 2, KGF—keratinocyte growth factor, EGFR—epithelial growth factor, RhoA—Ras homolog family member A, VEGF—vascular endothelial growth factor, TGF-β—transforming growth factor β, YAP—Yes-associated protein, STAT3—signal transducer and activator of transcription 3, IL—interleukin, DAMPs—damage associated molecular patterns, HSP—heat shock protein, ATP—adenosine triphosphate, TNF-α—tumor necrosis factor α, ROS—reactive oxygen species, miR—microRNAs, “+”—activation, “↑”—upregulation, “↓”—downregulation.

Section 3.3 focuses on regenerative medicine mediators, such as extracellular vesicles (EVs), stem cell derivates, miR-related alterations, and potential therapeutic targets related to these topics. Figure 2 represents the primary FETO-associated molecular alterations.

## 3. Synergic Treatment Options and Overcoming Complications

### 3.1. Treating PPROM and Preventing PB

Fetal surgery is continually striving toward becoming as minimally invasive as possible. One of the most significant challenges that persist, often referred to as “the Achilles’ heel of fetal surgery” [98], is the relatively high risk of PPROM, which frequently leads to preterm delivery and PB. A significant concern is that the amniotic sac does not easily reseal after surgery. Extensive research is being conducted on postoperative membrane healing. So far, studies involving tissue sealants, collagen plugs, and even platelet-rich plasma have produced promising results [99]. Table 1 outlines the clinical and experimental findings related to this subject.

### 3.2. Synergic Medical Therapies

Attempts to provide antenatal medical therapy for CDH are not new. Encountering pulmonary hypoplasia and hypertension are the two main points of prenatal CDH care. Various agents, including corticosteroids, retinoids, phosphodiesterase-5 (PDE5) inhibitors [110], tyrosine-kinase inhibitors, glucagon-like peptide-1 agonists, vitamins, and antioxidants, are currently being used or investigated for prenatal pharmacologic treatment [111]. It is important to note that most of these results are still experimental; only steroids and PDE5 inhibitors have progressed to the clinical phase. To the best of our knowledge, corticosteroids, retinoids, vitamin A, and PDE5 inhibitors were clinically or experimentally tested in conjunction with FETO.

Currently, corticosteroids are the only drugs that have been tested as a potential prenatal CDH therapy in a human randomized controlled trial [112]. Nowadays, they are widely applied to facilitate fetal lung maturation in women facing threatened preterm delivery, thereby reducing the respiratory morbidity and neonatal mortality [110]. The prenatal administration of betamethasone or dexamethasone is limited to two courses to avoid potential fetal and neonatal adverse effects [113]. In the FETO protocol, corticosteroids are also included. Patients undergoing the “plug” and “unplug” procedures are at risk for PB, and corticosteroids are administered in conjunction with these two interventions [15,16,114]. However, experimental studies reported mixed results regarding the effects of corticosteroids on TO.

Glucocorticoids enhance lung compliance via both surfactant-related and non-related mechanisms. They influence the enzymes involved in the surfactant synthetic pathway, resulting in increasing the synthesis of surfactant phospholipids and proteins [115].

It is important to understand that CDH-affected lungs do not function like normal ones. Experimental data indicate that the density of AE2 cells does not necessarily relate to the quantity or constituents of the surfactant. In lambs with CDH, those treated by TO and with prenatal glucocorticoids exhibited dysfunctional AE2 cells, decreased levels of bronchoalveolar phosphatidylcholine, and diminished lung tissue surfactants. Additionally, the synthesis and turnover of the endogenous surfactant is slow [115].

Surfactant non-related structural changes involve adjustments in the collagen-to-elastin ratio, a decrease in perilobar connective tissue, and reduced alveolar wall thickness, leading to an increase in aerated parenchyma [68].

The antenatal corticosteroid-related improvements in vasodilation response were observed; however, their impact on vascular proliferation is not completely understood. Histologically, they increase the number of distal vessels while reducing their muscularization [110].

Retinoids and derivations of vitamin A (retinol) are crucial for the normal development of the diaphragm and lungs. Retinoic acid (RA) is the active metabolite of retinol. It is a morphogen; however, if its levels are not carefully regulated, it can act as a teratogen [116]. Disruption of the retinoid signaling pathway is hypothesized to be a key element in the pathophysiology of CDH [117]. Nitrofen, an herbicide, inhibits the enzymes converting retinol to retinoic acid and the cellular uptake of retinol [118,119]. This herbicide has substantially impacted CDH research, as it is widely used in animal models to induce hypoplastic lung and diaphragmatic defects. It is also known that RA exerts its teratogenicity only when it is administered in the first trimester of pregnancy. In contrast, FETO is effectuated at the end of the second one. In vitro, RA can stimulate VEGF signaling, which promotes angiogenesis. Thinning of the muscular wall of pulmonary arterioles eases pulmonary hypertension [93]. An antenatal treatment that combined RA with TO was tested in several models.

Sildenafil is a selective PDE5 inhibitor, which degrades cyclic guanosine monophosphate (cGMP). PDE5 is abundantly expressed during fetal development in the pulmonary arteries, acting as a key regulator of circulation [120]. Experimental animal models of newborns with chronic pulmonary hypertension revealed increased secretion of vasoconstrictors and impaired endothelial release of NO. The elevated activity of PDE5 may also contribute to this circumstance [121]. Maternal sildenafil and TO were tested together in a rabbit model [122]. Table 2 summarizes the main ideas of such studies with mixed treatments, and the results of these studies varied.

### 3.3. Role of EVs and Regenerative Medicine in CDH Treatment

EVs are a heterogenous group of cell-derived particles enclosed by lipid bilayers. Various cell types secrete EVs into extracellular fluids, and they carry specific markers and content associated with their cell of origin. EVs are crucial for intercellular communication, transporting proteins, lipids, and nucleic acids to their intended target cells [132,133].

EVs have diverse clinical applications, particularly in regenerative medicine, where stem-cell-derived EVs promote tissue repair. They have also been studied for their diagnostic potential, with biomarkers leading to noninvasive tests. Additionally, they can deliver targeted therapeutics, overcoming traditional delivery limitations. However, challenges remain, such as optimal isolation methods, the stability of EV cargo, and evolving regulatory issues [134].

Recent reports on CDH research show that amniotic-fluid-stem-cell-derived EVs (AFSC-EVs) promote normal development in pulmonary hypoplasia rodent models. These EVs enhance epithelial cell differentiation and branching morphogenesis at the pseudoglandular stage in fetal rat lung explants. The differentiation of lipofibroblasts and the number of alveoli are also increased at the alveolar stage. These effects are related to their miR cargo, which has regenerative effects on lung development [135,136]. In a recent prospective study on humans, different miR expression levels were found between CDH and control samples: 148 miRs were identified to be upregulated and 36 downregulated in CDH infants’ blood. Dysregulated miRs were associated with transcription regulation, protein binding, and signaling pathways linked to pulmonary hypertension and hypoplasia. Based on a random forest analysis of maternal blood, miR-942-3p, miR-7850-5p_L-1R+2, and miR-197-3p effectively distinguished CDH survivors from non-survivors. These circulating miRs show promise as potential biomarkers for predicting outcomes. Additionally, miRs extracted from the infants’ blood may serve as possible therapeutic targets in key pathways related to pulmonary hypertension and hypoplasia [137]. In tracheal aspirates of FETO-survived fetuses with significant lung growth, there were found higher levels of miR-200b compared with those who did not respond to TO [136]. Table 3 presents several human and experimental studies on EVs and regenerative medicine.

### 3.4. Novel Ways to Deliver Mediators

In a fetal sheep CDH model, bisguanidiniumtren-cholesterol/dioleoyl-phosphatidylethanolamine (BGTC/DOPE) cationic liposomes were tested to deliver KGF into the fetal airways. When KGF transfection was combined with TO, it not only corrected the CDH-associated lung hypoplasia and reduced radial alveolar count as TO alone did but also increased the surfactant protein B synthesis [81].

Intravenously delivered cationic poly(amine-co-ester) nanoparticles loaded with miR-200b were successfully tested in a rat model. miR-200b induces epigenetic changes in the TGF-β pathway, activating TGF-β/SMAD signaling and increasing branching morphogenesis [147]. Similarly, microinjection-delivered functionalized IgG-conjugated nanoparticles (IgG-nanoparticles) were tested in vivo on rat lung explants to study prenatal therapy [148].

### 3.5. Surgical- and Technique-Related Modifications of FETO

Every surgical intervention has its limitations, approach, and technique-related disadvantages. Even in the case of FETO, which is a so-called relatively simple intervention, there is room for technique-related improvements.

Biotechnological engineering plays an important role in developing new technologies. One such invention is the “Smart-TO” balloon, which enables noninvasive and easy disconnection through a magnetic valve activated by the magnetic fringe field of a magnetic resonance imaging (MRI) scanner. This design eliminates the need for a second surgical intervention. The device was first tested in a nonhuman primate model [149] and has since been successfully translated into the clinical phase [150].

There are even more benefits of this technique. The mortality and morbidity associated with FETO are partially related to airway reestablishment (plugging and unplugging) issues. The timing of these sequences might need to be reconsidered with this new technique. Unplugging could potentially be delayed since there are no technical limitations in reversing the procedure. Additionally, FETO can be conducted later in pregnancy, which prevents subsequent PB without reducing the overall duration of occlusion. There have also been reported benefits in terms of maternal compliance and reduced hospitalization time [149]. Further studies are needed to explore these findings.

Another technique to overcome the unplugging intervention is to use a balloon-substituting material, which degrades and frees the trachea after it fulfills its purpose. For this, a fibrin glue gel plug was tested in an ex vivo rabbit model. However, it proved effective only for a short period and did not provide sufficient sealing, resulting in increased airway resistance [151]. Another study using a temporary fibrin plug showed a moderate but measurable positive effect on alveolar development and increased adventitial thickness [152]. Other hydrogels with beneficial properties were also evaluated, but these tests were limited to in vitro experiments [153].

A dynamic TO device that preserves changes in fetal breathing pressure and fluid flow was also studied in a lamb model of CDH. The data show that this device resulted in more physiological lung morphometrics and function than the complete occlusion controls [154]. Additionally, a reversible thermally actuated polymer valve occlusion tool was characterized by its use in dynamic TO [155].

Gastroschisis may improve pulmonary outcomes in CDH by serving as a “pop-off valve” that decompresses the thorax, thereby reducing the pressure on the developing lungs. Several syndromes (Fryns, Beckwith–Wiedemann syndrome, Cantrell’s pentalogy), and rarely in nonsyndromic cases, can combine/involve both malformations and are associated with better respiratory outcomes [156].

In the era of open fetal surgery for treating CDH, multiple reports have indicated that the iatrogenic creation of gastroschisis may have beneficial effects as a palliative prenatal intervention [157,158]. Experimental studies showed that pulmonary hypoplasia in newborn rabbits is less severe when gastroschisis or tracheal ligation is performed simultaneously [159].

One notable case report highlights a combined minimally invasive prenatal intervention. During the FETO procedure, herniated bowels from the fetal chest were partially removed through fetoscopic laparoschisis (FETO-LAP) in a fetus with severe left-sided congenital CDH. This approach was well tolerated and regarded as a life-saving therapeutic option [160].

An alternative and exciting surgical approach was tested on a left CDH fetal sheep model. This method involved selectively occluding the left main bronchus to restore the left lung only, reduce the herniation of the organs on the left, and recuperate space in the thoracic cavity necessary for lung development. Despite this, the right wet lung-to-body weight ratio remained low, while the left lung overgrew, leading to a mediastinal shift. Similarly, it did not reduce the amount of herniated viscera [161].

## 4. Conclusions

CDH remains a complex condition with significant challenges in neonatal mortality and morbidity despite modern multidisciplinary approaches. FETO has emerged as a crucial intervention, promoting lung development and enhancing outcomes for affected fetuses. However, its benefits must be weighed against risks, including complications for maternal and fetal health. Before obtaining parental consent, it is essential to discuss these risks and potential outcomes with their healthcare team to make an informed decision. Ongoing studies are needed to understand the mechanisms behind FETO’s effectiveness. Future efforts should integrate molecular-targeting therapies and regenerative medicine alongside advancing surgical methodologies.

## Figures and Tables

**Table 1:** Summary of the current experimental and clinical literature on membrane repair preventing PPROM.

Human Studies	Conclusions
Tchirikov et al., 2017 [100]	Small iatrogenic amniotic membrane defects successfully treated by laser coagulation technique.
Chmait et al., 2017 [101]	“Amniopatch”: higher GA at delivery and higher perinatal survival rates in almost two-thirds of cases.
Sung JH et al., 2017 [102]	In the iatrogenic PPROM group, the “Amniopatch” had a 36.4% success rate. Larger volumes of amniotic fluid before the procedure were key predictors of procedural success.
**Experimental Studies**	**Conclusions**
Kondoh et al., 2021 [103]	Intracervical elastomeric sealant (fibrin glue) demonstrated good fluid leakage prevention in an ex vivo model.
Devaud et al., 2021 [104]	Histoacryl^®^ and Glubran2^®^ tissue adhesives with umbrella-shaped receptors successfully sealed membrane defects in a sheep model.
Byju et al., 2022 [105]	The percutaneously delivered, resorbable “ChorioAnchor” device can secure the chorioamniotic membranes to the uterine wall—fulfills its engineering specifications during the initial phases of implantation.
Micheletti et al., 2022 [106]	In vivo and ex vivo sheep models: a fetoscopic applied semirigid silicone-hydroxypropyl methylcellulose patch sealed membrane defects.
Avilla-Royo et al., 2022 [107]	Mussel-inspired biomimetic glue had promising properties for the sealing of fetal membrane defects in an ovine model.
Devaud et al., 2023 [108]	Cyanoacrylate-based sealing patches led to a watertight seal at 10 or 24 days post-treatment in an ovine model.
Bergh et al., 2024 [109]	Suturing device tested ex vivo and in vivo: anchored amniotic membranes to the underlying myometrium.

Abbreviation: GA—gestational age.

**Table 2:** Summary of studies on antenatal medical therapies for CDH related to FETO.

Corticosteroids	Conclusions
Bratu et al., 2001 [68]	Reversible tracheal occlusion (TO) and prenatal betamethasone led to similar pulmonary architectures to the controls and offered no added benefit in terms of surfactant production.
Bratu et al., 2001 [123]	Reversible TO and antenatal glucocorticoids prevented the thinning of the small pulmonary arteries and enhanced the lung growth and structural maturity.
Davey et al., 2006 [124]	Glucocorticoids reduced the lung liquid volume during TO, which also increased the AE2 cell density and surfactant protein mRNA expression.
Davey et al., 2006 [125]	TO plus glucocorticoid or surfactant significantly improved the respiratory gas exchange, lung compliance, and ventilatory efficiency index. The total lung capacity was normalized only when the glucocorticoids and surfactant were administered together.
Davey et al., 2007 [126]	TO and prenatal glucocorticoid treatments reduced the medial pulmonary arteriole hypertrophy by 19% in a severe congenital diaphragmatic hernia (CDH) fetal sheep model.
Mayer et al., 2008 [127]	Prenatal betamethasone inhibited lung proliferation in TO-treated nitrofen-induced CDH rat fetuses.
Roubliova et al., 2009 [128]	TO and betamethasone had a cumulative effect on reducing peripheric muscularization.
**Retinoids**	**Conclusions**
Schmidt et al., 2016 [129]	TO and retinoic acid (RA) together had no additional benefit in reducing the median pulmonary arteriole wall thickness or in increasing the VEGF and its receptors.
Delabaere et al., 2017 [130]	Liposomes and Miglyol could be used as vehicles for delivering RA into fetal airways. Tracheal RA opposed the effects of TO and improved the surfactant production in rabbit fetuses with normal lungs.
Delabaere et al., 2018 [131]	TO and RA had synergic effects on vascular measurements, proportional medial thickness, and endothelin-1 receptor type-A gene expression, and restored pneumocyte differentiation.
**Phosphodiesterase Inhibitors**	**Conclusions**
Russo et al., 2022 [122]	TO and maternal sildenafil had complementary effects on the vascular and parenchymal lung development. They also counteracted the reduced gene expression of VEGF and surfactant proteins A and B induced by TO (tested in a rabbit model).

Abbreviations: TO—tracheal occlusion, CDH—congenital diaphragmatic hernia, RA—retinoic acid.

**Table 3:** Summary of current research on CDH focusing on EVs and regenerative medicine.

EVs	Conclusions
Pereira-Terra et al., 2015 [136]	Fetal CDH lungs presented elevated expressions of miR-10a and miR-200b; miR-200b was elevated at balloon removal and in FETO survivors; this miR inhibited the TGF-β-induced SMAD signaling.
Monroe MN et al., 2020 [138]	Mesenchymal-stem-cell-derived extracellular vesicles (MSC-EVs) reversed extracellular matrix (ECM) remodeling in the CDH pulmonary vasculature: bolstered structural aspects of the pulmonary artery ECM and mitigated pathological disorganization, as exhibited by an increased medial wall thickness and stiffness.
Zhaorigetu S et al., 2020 [139]	MSC-EV treatment improved the cellular responses, including key endothelial dysfunction proteins in a nitrofen-induced CDH model. In vivo, MSC-EV exposure enhanced the pulmonary artery contractile response and reduced the pulmonary vascular dysfunction.
Fabietti I et al., 2021 [140]	Higher extracellular vesicle (EV) counts in the amniotic fluid of non-survivors and tracheal fluid collected during TO reversal indicated established pro-inflammatory lung reactivity in utero, potentially linked to poorer postnatal outcomes. The regulation of EV-derived miR-223-3p, miR-379-5p, miR-503-5p, and miR-889-3p was related to postnatal survival. Their target genes were possibly associated with altered lung function.
Antounians L et al., 2021 [141]	Overexpression of miR17-92 cluster in amniotic-fluid-stem-cell-derived EVs (AFSC-EVs) was observed in EV-treated primary lung epithelial cells.
Khalaj K et al., 2022 [135]	AFSC-EVs improved the airspace density and branching morphogenesis, and enhanced the alveolar cell markers during canalicular and saccular stages; they also restored the cell markers of ciliated epithelial, club, and pulmonary neuroendocrine cells at the saccular stage to control levels, along with lipofibroblasts and PDGFRA+ markers.AFSC-EVs transferred the miR-17-92 cluster to rescue branching morphogenesis and partially restored autophagy.
Matsuo S et al., 2024 [142]	AFSC-EV-derived miRs could prenatally predict severe CDH cases with a high accuracy; changes in these miR profiles could reflect the status of the lungs.
Figueira R et al., 2024 [143]	The administration of AFSC-EVs led to improvements in lung mechanics (resistance, elastance, compliance, tissue damping), as well as collagen deposition and branching morphogenesis.
Doktor F et al., 2024 [144]	The administration of AFSC-EVs facilitated lung growth (reduced mean linear intercept), vascularization (increased Enos and Cd31), and decreased inflammation (TNF-α, IL-1b).
Antounians L et al., 2024 [145]	AFSC-EVs injected into rats with CDH enhanced lung branching and epithelial differentiation; this treatment also reversed the inflammatory response with macrophage enrichment exhibited by these lungs.
Doktor F et al., 2025 [146]	AFSC-EV administration facilitated lung branching and patterning of airway progenitor cells, partly via miR-93-5p release. It blocked SMAD 7, leading to pSMAD2/3 upregulation and TGF-β signaling restoration. Antagomir 93-5p-treated oligohydramnios lungs showed different results: decreased TGF-β signaling and branching morphogenesis.

Abbreviations: MSC-EVs—mesenchymal-stem-cell-derived extracellular vesicles, ECM—extracellular matrix, EVs—extracellular vesicles, AFSC-EVs—amniotic-fluid-stem-cell-derived extracellular vesicles.

## Data Availability

Not applicable.

## Abbreviations

AE2	Alveolar epithelial type II (cells)
AFSC-EVs	Amniotic-fluid-stem-cell-derived extracellular vesicles
AKI	Acute kidney injury
ATP	Adenosine triphosphate
CDH	Congenital diaphragmatic hernia
cGMP	Cyclic guanosine monophosphate
DAMPs	Damage-associated molecular patterns
ECM	Extracellular matrix
ECMO	Extracorporeal membrane oxygenation
EGFR	Epithelial growth factor
ERK	Extracellular signal-regulated kinase
EVs	Extracellular vesicles
EXIT	Ex utero intrapartum therapy
FETO	Fetoscopic endoluminal tracheal occlusion
FETO-LAP	Fetoscopic laparoschisis
Fgf10	Fibroblast growth factor 10
FgfR2	Fibroblast growth factor receptor 2
GA	Gestational age
GERD	Gastroesophageal reflux disease
GI	gastrointestinal
HSP	Heat shock protein
IGF	Insulin-like growth factor
IL	Interleukin
KGF	Keratinocyte growth factor
miR	MicroRNA
MRI	Magnetic resonance imaging
MSC-EVs	Mesenchyomal-stem-cell-derived extracellular vesicles
mTOR	Mammalian target of rapamycin
MWT	Medial wall thickness
NO	Nitrogen oxide
PB	Preterm birth
PCNA	Proliferating cell nuclear antigen
PDE	Phosphodiesterase
PDGF	Platelet-derived growth factor
PPROM	Preterm prelabor rupture of membrane
RA	Retinoic acid
RhoA	Ras homolog family member A
ROS	Reactive oxygen species
Spry2	Sprouty homolog 2
STAT3	Signal transducer and activator of transcription 3
TGF-β	Transforming growth factor β
TNF-α	Tumor necrosis factor-α
TO	Tracheal occlusion
VEGF	Vascular endothelial growth factor
YAP	Yes-associated protein
